# Staged reconstruction of severe perineal destruction with open pelvic and femoral fractures: a 5-year follow-up case report

**DOI:** 10.1097/RC9.0000000000001120

**Published:** 2026-10-14

**Authors:** Kehai Mei, Ye Xu, Lizhi Yu, Jinzhan Sun

**Affiliations:** a Department of Orthopedic Trauma, Binzhou Central Hospital, Binzhou, China; b Department of Orthopedic Spine Surgery, Binzhou People’s Hospital, Binzhou, China

**Keywords:** case report, damage control orthopedics, NPWTi, open pelvic fracture, perineal injury

## Abstract

**Introduction and importance:**

Open pelvic fracture with perineal destruction and an open femoral fracture is uncommon and carries a high risk of infection and functional loss. Concurrent management of the pelvic ring, femur, and perineal soft tissues is particularly demanding.

**Presentation of case:**

A 36-year-old man sustained a high-energy industrial crush injury with an open pelvic fracture, an open femoral shaft fracture, and a heavily contaminated perineal wound. After resuscitation, external fixation, and exploration, colostomy and urinary diversion were omitted. Infection was controlled with serial debridement and anus-sparing negative-pressure wound therapy with instillation (NPWTi), followed by skin grafting. The pelvic ring remained stable after external fixation. Femoral reconstruction with internal fixation and an external fixator was performed on day 84. Delayed union at 14 months was managed with staged dynamization. At 51 months, he had returned to work; the Harris Hip Score was 87 and the Majeed Pelvic Score was 86. Radiographs at 60 months showed maintained fracture union and bone remodeling.

**Clinical discussion:**

With an intact anorectum, anus-sparing NPWTi was used without fecal diversion. Staged fixation balanced infection risk and stability. Dynamization for delayed union was followed by callus formation and union. This approach does not replace fecal diversion when the anorectum is injured.

**Conclusion:**

A staged approach to damage control, infection control, and delayed reconstruction may help selected patients with severely contaminated open pelvic and femoral fractures to achieve favorable outcomes.

## Introduction

Open pelvic fractures are difficult to treat because of hemorrhage, contamination, and instability. Concurrent perineal destruction and an open femoral fracture further complicate care. Fecal diversion is often considered for severe perineal injury, but an intact anorectum may allow an alternative strategy if contamination can be controlled. This case report has been prepared in line with the SCARE 2025 checklist^[^1^]^.

HIGHLIGHTS

- An intact anorectum allowed anus-sparing negative-pressure wound therapy with instillation without fecal diversion.
- Delayed femoral fixation, on day 84, followed infection and soft-tissue control.
- Staged dynamization of the frame was followed by union at 23 months.
- At 51 months, the Harris Hip Score was 87 and the Majeed Pelvic Score was 86.
- Five-year radiographs showed maintained alignment, union, and remodeling.

## Case presentation

### Initial presentation and injury assessment

A 36-year-old Chinese man was admitted after a crush injury on 18 August 2021 when he fell into an industrial mixer. The patient had no significant past medical history, no known drug allergies, and no relevant family or social history. He was a non-smoker and lived independently. On arrival, he was hemodynamically unstable (blood pressure 80/51 mmHg, heart rate 104 beats per minute, respiratory rate 27 breaths per minute, oxygen saturation 99%, Glasgow Coma Scale score of 15). Examination revealed a massive perineal soft-tissue defect approximately 30 cm × 10 cm extending to the right proximal thigh (approximately 50% of thigh circumference) (Fig. 1A), as well as extensive contusion and skin loss over the left gluteal and lower back regions measuring approximately 40 cm × 20 cm. The wounds were contaminated with soil and foreign material; transected muscle ends and exposed femoral fracture fragments were visible. Distal pulses were palpable and symmetrical. Distal motor and sensory function was preserved. After initial resuscitation, he was transferred to an orthopedic ward on day 2.

**Figure 1.**
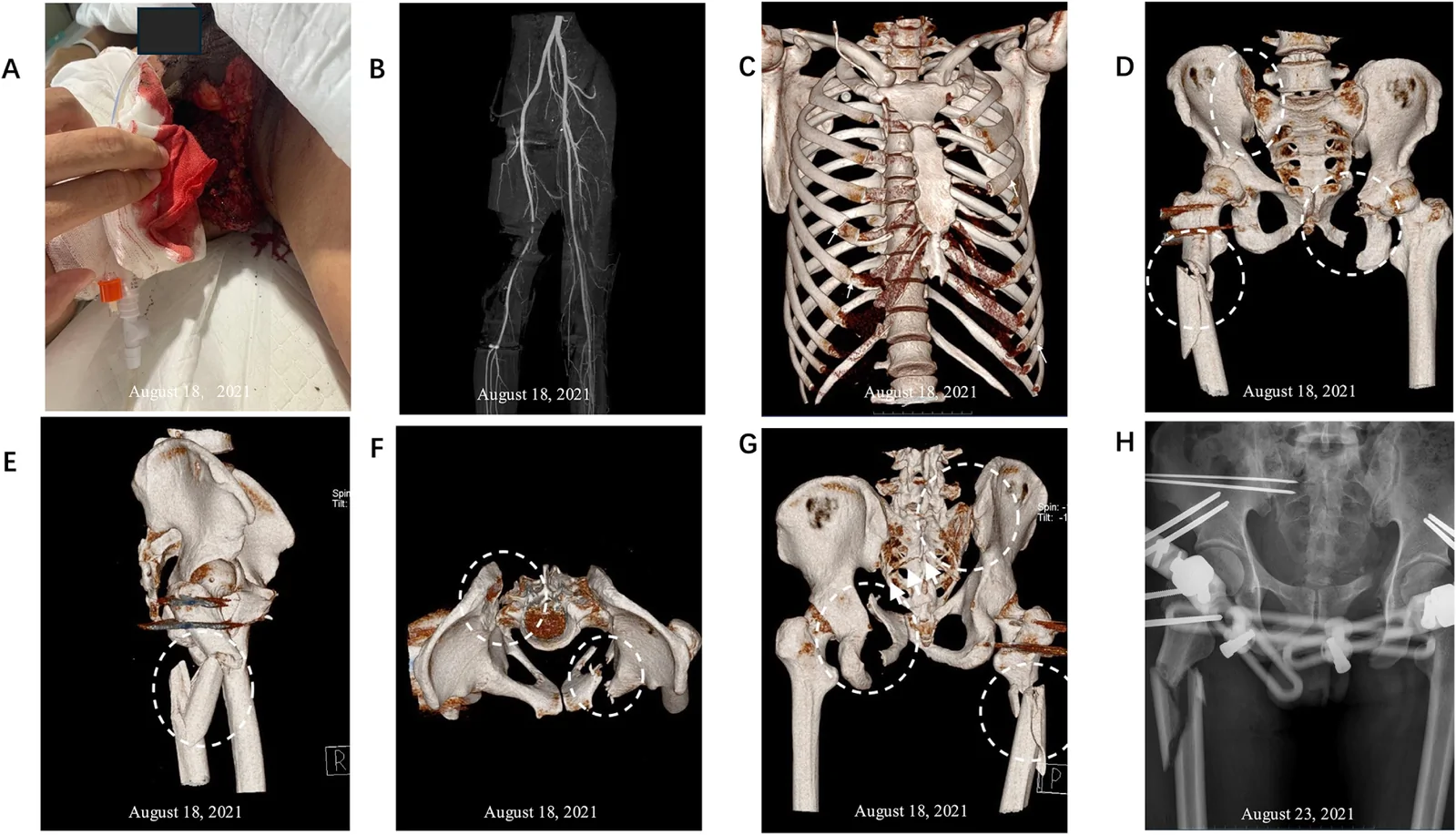
Initial evaluation after admission. (A) Clinical photograph of the extensive perineal wound. (B) CT angiography of both lower extremities showing intact vascular structures. (C) Three-dimensional chest CT reconstruction showing multiple rib fractures (arrows). (D–G) Three-dimensional CT reconstructions of the pelvis and femur. Dashed circles indicate right sacroiliac joint dislocation, left superior and inferior pubic rami fractures, and right femoral fracture (monolateral frame; Aidite Technology, Suzhou, China; WGDZa Φ32 × 250). (H) Anteroposterior radiograph after temporary external fixation. A pelvic external fixator was applied on day 5 (Yuelai Medical Devices, Zhangjiagang, China; GP-A).

### Imaging findings and injury classification

Angiography showed no major vascular injury (Fig. 1B). Emergency CT showed multiple rib fractures (Fig. 1C) and a complex pelvic–femoral injury (Fig. 1D–G). The pelvic ring was a Young–Burgess CM injury with a predominant VS component, Tile C1 (AO/OTA 61-C1.2): complete right sacroiliac disruption with cephalad displacement of the right hemipelvis, plus left superior and inferior pubic rami fractures. A nondisplaced transverse sacral fracture was recorded separately. A single Faringer zone I perineal wound extended to the posterior right thigh and communicated with the pelvic and femoral fracture ends. After debridement, the shared wound was classified as Gustilo–Anderson IIIB. The femur was an open diaphyseal fragmentary wedge fracture with two intact butterfly fragments (AO/OTA 32-B3).

### Initial management and contamination control

On admission, hypovolemia was corrected with 7.5 units of packed red blood cells and 1590 mL of plasma, plus broad-spectrum antibiotics and tetanus prophylaxis. After relative stability was restored, an orthopedic, urological, and colorectal team explored the wound. The sciatic nerve was in continuity. The penis, testes, urethra, rectum, and anal canal were intact; because the anorectum was intact and the perianal wound could be isolated, neither cystostomy nor colostomy was performed. The femur was stabilized with a monolateral external fixator. An anus-sparing NPWTi dressing was placed in the deep perineal–femoral cavity and instilled with about 2000 mL/day of 0.9% saline. On day 5, a pelvic external fixator was applied, the right sacroiliac joint was reduced under fluoroscopy, and two Kirschner wires were inserted (Fig. 1H).

### Serial debridement and infection-control strategy

Serial debridement and NPWTi were performed five times in the first 16 days (Supplemental Digital Content Table S1, available at: https://links.lww.com/IJSCR/A189), at −125 mmHg, with dressing changes every 3–4 days. On day 7, diarrhea soiled the wound; however, the NPWTi seal was maintained. Empiric intravenous piperacillin-tazobactam, 4.5 g every 6 hours, was started on admission. After a wound-swab culture grew *Escherichia coli*, therapy was changed to intravenous ceftriaxone, 1 g twice daily, according to susceptibility testing. Antibiotics were continued for 36 days and stopped when the wound had largely healed. Local inflammation, white blood cell count, C-reactive protein, procalcitonin, radiographs, and serial wound cultures were used to monitor infection (Table 1). Once the wound bed was stable, split-thickness skin grafts were applied to residual defects over the left buttock and back, with partial graft take (Fig. 2A).

**Figure 2.**
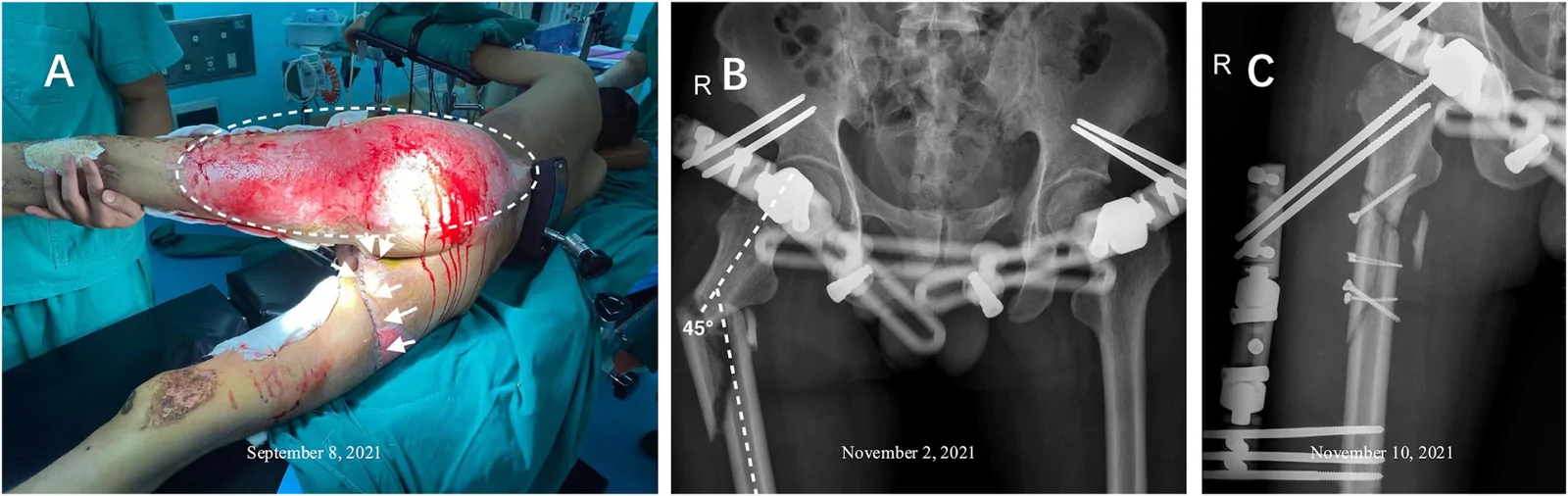
Soft-tissue coverage and definitive femoral reconstruction. (A) Intraoperative photograph during skin grafting of the left buttock and back. Dashed circle indicates residual skin defect; arrow indicates healed perineal wound. (B) Anteroposterior radiograph showing lateral angular deformity before reconstruction. (C) Anteroposterior radiograph after reconstruction with limited internal fixation and a monolateral external fixator (Aidite Technology, Suzhou, China; WGDZa Φ32 × 250).

**Table 1:** Serial changes in inflammatory markers from admission to definitive femoral reconstruction.

Date	WBC (×10^9^/L)	Neutrophil count (×10^9^/L)	Neutrophil (%)	ESR (mm/h)	CRP (mg/L)	PCT (ng/mL)
18/08/2021	19.06	13.70	69.9	2	1.06	–
19/08/2021	7.29	6.74	92.3	–	89.61	–
20/08/2021	7.91	7.15	90.4	–	223.14	–
21/08/2021	6.46	5.74	88.9	–	195.70	–
22/08/2021	4.87	4.03	82.8	97	–	–
23/08/2021	2.90	2.14	73.6	–	126.16	0.72
24/08/2021	6.57	5.99	91.3	28	–	–
25/08/2021	8.37	6.66	79.6	33	151.05	1.21
07/09/2021	5.79	4.40	75.9	48	–	–
18/10/2021	5.49	2.37	43.1	38	6.64	–
11/11/2021	8.19	6.17	75.3	8	–	–

WBC, white blood cell count (reference range, 3.50–9.50 × 10^9^/L); neutrophil count (1.80–6.30 × 10^9^/L); neutrophil percentage (40.00–75.00%); ESR, erythrocyte sedimentation rate (0–15 mm/h); CRP, C-reactive protein (0–6.00 mg/L); PCT, procalcitonin (0–0.05 ng/mL); –, not measured.

### Definitive femoral reconstruction

Serial radiographs showed that the pelvic fracture lines remained reduced without progressive displacement, and the pelvic ring remained clinically and radiographically stable, so further pelvic reconstruction was not required. Femoral alignment was lost; preoperative radiographs showed approximately 45° of lateral angulation of the right femur after initial external fixation (Fig. 2B). Definitive reconstruction was deferred until infection control and soft-tissue stability were considered adequate, based on the following findings: complete healing of the perineal wound and skin grafts without inflammation or discharge, and no laboratory evidence of ongoing or recrudescent infection (Table 1). After these criteria were met, reconstruction was performed on day 84. Under general anesthesia in the left lateral decubitus position, excess callus and a large lateral butterfly fragment were addressed. The fragment was reduced and fixed with one oblique lag screw and two titanium cables. Sclerotic bone ends were resected, the medullary canal was reopened, and a second lag screw was used to restore alignment. A residual posteromedial fragment was left untreated. The posterior bone defect was pulse-lavaged, packed with allogeneic bone, and covered with vancomycin powder. Stability was achieved with limited internal fixation using screws and titanium cables combined with a unilateral external fixator (Fig. 2C). The patient remained non-weight-bearing for 6 weeks and then progressed to partial weight-bearing according to radiographic and clinical progress (Fig. 3A). The pelvic external fixator was removed at 3 months.

**Figure 3.**
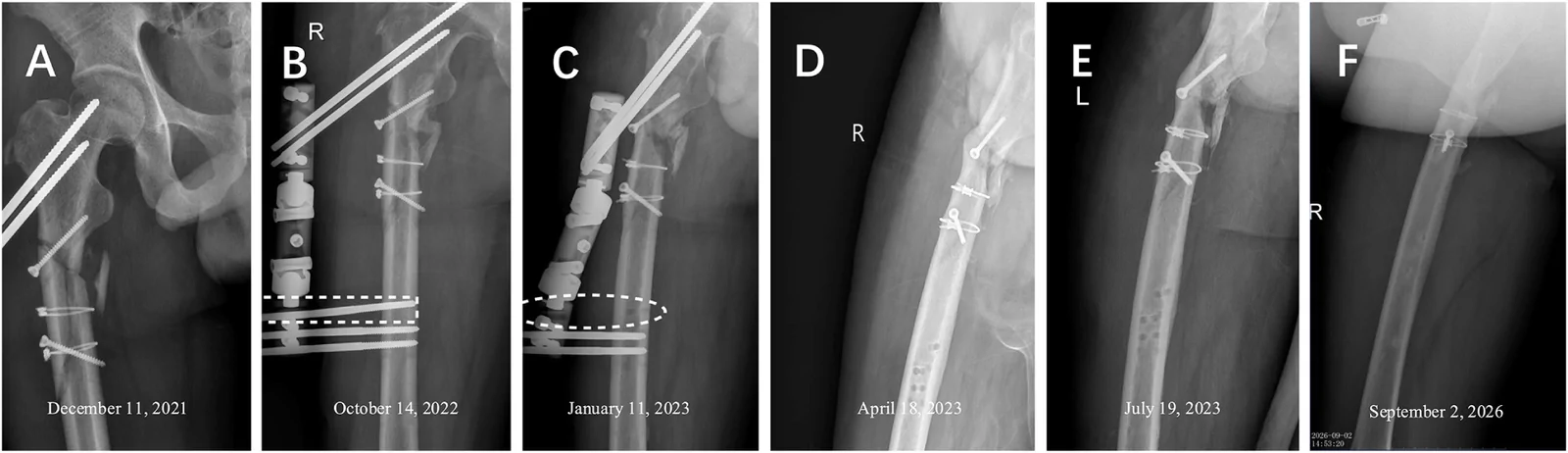
Radiographic progression after reconstruction and dynamization. (A) Lateral radiograph of the femur at 4 months after injury. (B) Lateral radiograph of the femur at 14 months after injury. The dashed line indicates the site of the Schanz pin to be removed. (C) Lateral radiograph of the femur at 17 months after injury. The dashed circle marks the former pin tract. (D) Lateral radiograph of the femur at 20 months after injury. (E) Lateral radiograph of the femur at 23 months after injury. (F) Lateral radiograph of the femur at 60 months after injury.

### Management of delayed union and dynamization

At 14 months post-injury, a persistent fracture gap with limited callus raised concern about delayed union (Fig. 3B). The distal Schanz pin closest to the fracture was therefore removed. The frame was loosened at 16 months and removed at 17 months (Fig. 3C). There was no refracture and no new pain. Callus increased thereafter (Fig. 3D), with radiographic and clinical union by 23 months (Fig. 3E).

### Long-term follow-up and functional outcomes

The patient was discharged on day 117. At approximately 51 months after injury, the Harris Hip Score was 87 and the Majeed Pelvic Score was 86. The patient had returned to work as a loader without formal restrictions and was able to cycle. Sexual function was preserved, although endurance was partly limited by occasional right hip pain and a mild limp. There was no recurrent or implant-related infection (Supplemental Digital Content Figure S1, available at: https://links.lww.com/IJSCR/A190 and Supplemental Digital Content Figure S2, available at: https://links.lww.com/IJSCR/A191). The patient reported that he was satisfied with the overall outcome and felt that his current functional status was close to his pre-injury level. At the latest radiographic follow-up on 2 September 2026, approximately 5 years after injury, radiographs showed maintained alignment, fracture union, remodeling along the mechanical axis, and increased bone density (Fig. 3F). The clinical course is summarized in Table 2.

**Table 2:** Timeline of events in chronological order.

Time after injury	Event
Day 0	Resuscitation and transfusion; pelvic binder application; MDT-guided exploration (anorectum, urethra and sciatic nerve intact); debridement; femoral external fixation; anus-sparing NPWTi (Aidite Technology, Suzhou, China; AD-1-15 × 5 × 1, PVA); no colostomy or cystostomy
Day 5	Repeat debridement and NPWTi replacement; pelvic external fixation and reduction of the right sacroiliac joint
Day 5–84 after injury	Serial debridement, NPWTi, skin grafting and maintenance of the external fixators until the wound and infection markers were stable.
Day 84	Femoral reconstruction: limited internal fixation with screws and titanium cables combined with a unilateral external fixator; non-weight-bearing for 6 weeks postoperatively
3 months after injury	Removal of the pelvic external fixator
14 months after injury	Dynamization was performed by removing the Schanz pin closest to the fracture site among the three distal Schanz pins.
16 months after injury	Loosening of the external fixator components
17 months after injury	Removal of all external fixator components
20 months after injury	Follow-up radiographs demonstrated progressive callus formation across the fracture site, and functional rehabilitation was continued.
23 months after injury	Follow-up radiographs confirmed progressive fracture healing and satisfactory union at the fracture site.
51 months after injury	Functional evaluation showed satisfactory outcomes, with a Harris Hip Score of 87 and a Majeed Pelvic Score of 86.
60 months (approximately 5 years) after injury	Latest radiographic follow-up showed maintained fracture union with remodeling and increased bone density.

## Discussion

Early management prioritized hemorrhage control, physiological stabilization, and temporary external fixation^[^2–4^]^. Because multidisciplinary exploration confirmed an intact anorectum and that the perianal wound could be effectively isolated, fecal diversion was not performed. An anus-sparing NPWTi dressing limited fecal contamination and facilitated irrigation and drainage of the communicating perineal–femoral cavity. Despite intermittent wound soiling from diarrhea, serial debridement, culture-guided antibiotics, and NPWTi at −125 mmHg were associated with progressive wound improvement and fulfillment of the predefined infection-control criteria^[^5,6^]^. Femoral reconstruction was therefore deferred until soft-tissue stability was established.

A more extensive internal fixation construct was avoided because the recently healed but previously contaminated soft-tissue envelope had limited reserve; intramedullary nailing or long-plate fixation would also have introduced a greater internal implant burden or required more extensive soft-tissue exposure. Limited internal fixation combined with an external fixator was selected to balance mechanical stability, soft-tissue preservation, and infection risk in the previously contaminated field^[^7,8^]^. During follow-up, a persistent fracture gap and limited callus formation suggested insufficient mechanical stimulation. Staged dynamization was subsequently performed and was followed by progressive callus formation and union^[^9,10^]^.

Previous reports of severe open pelvic and perineal injuries have emphasized fecal diversion and staged soft-tissue management, particularly when anorectal injury is present^[^11,12^]^. In this case, multidisciplinary exploration confirmed an intact anorectum, allowing fecal diversion to be avoided while contamination was controlled with anus-sparing NPWTi and serial debridement. The subsequent delayed femoral reconstruction and staged dynamization further illustrate how fixation strategy and timing may be adapted to the evolving soft-tissue condition and fracture-healing response.

This case illustrates three points: anus-sparing NPWTi without prophylactic diversion may be considered when the anorectum is intact and the wound can be isolated; delayed, individualized fixation may balance infection risk and stability in contaminated fractures; and staged dynamization may aid healing when a delayed union is accompanied by limited callus. These measures do not replace diversion in hollow-viscus injury, and a single case cannot prove superiority.

Limitations include the single-patient design, the inability to isolate the effect of each staged step, and the reliance on clinical assessment of function in a single patient.

## Conclusion

Early damage control, staged cavity drainage, delayed definitive fixation, and staged dynamization of the fixator may help selected patients with severely contaminated open pelvic and femoral fractures achieve a favorable outcome.

## Artificial Intelligence (AI) Statement

Generative AI tools were used during manuscript preparation and revision from June to September 2026. ChatGPT (OpenAI, GPT-5.5) and Grok (xAI) were used for English-language editing, translation, grammar correction, wording refinement, and manuscript formatting. They were not used for patient diagnosis, treatment decisions, clinical management, data collection or analysis, or interpretation of clinical findings. Only de-identified manuscript and clinical information were provided; no directly identifying patient information was entered. The AI tools were accessed as cloud-based services without custom training or fine-tuning. All AI-assisted content was reviewed and verified by the authors against the original clinical records and source materials, with inaccurate or inappropriate suggestions revised or discarded. The authors take full responsibility for the accuracy, integrity, and final content of the manuscript and acknowledge the potential for AI-generated errors, bias, and inappropriate outputs. No AI-generated clinical images or figures were used in the manuscript. AI use did not influence patient management or the clinical conclusions of this report.
